# Nanocarrier-mediated targeting of NF-κB and JAK/STAT signaling pathways in hepatocellular carcinoma: mechanisms and therapeutic strategies

**DOI:** 10.1186/s13046-026-03715-5

**Published:** 2026-05-04

**Authors:** Muhammad Azhar, Yang Duan, Muhammad Ahmad, Haichuan Wang

**Affiliations:** 1 Division of Liver Surgery, Department of General Surgery and Laboratory of Liver Surgery, West China Hospital, Sichuan University, No. 37 Guo Xue Xiang, Chengdu, 610041 Sichuan China; 2 Department of Urology, Institute of Urology, West China Hospital, Sichuan University, Chengdu, P.R. China

**Keywords:** Hepatocellular carcinoma, NF-κB signaling, JAK/STAT pathway, Nanotechnology-based drug delivery, Targeted cancer therapy

## Abstract

Hepatocellular carcinoma (HCC) remains a major global cause of cancer-related deaths. HCC development, immune evasion, and treatment resistance are significantly influenced by the aberrant activation of the NF-κB and JAK/STAT signaling pathways. The review provides a thorough and critical analysis of recent developments in nanocarrier-mediated targeting of NF-κB and JAK/STAT pathways in HCC. It demonstrates the molecular interactions between various pathways and their implications for inflammation, angiogenesis, hepatocarcinogenesis, and resistance to both immunotherapy and chemotherapy. A variety of nanoplatforms, such as polymeric nanoparticles, lipid-based systems, inorganic nanomaterials, and biomimetic carriers, are designed to enhance the delivery of small-molecule inhibitors, nucleic acids, and combination therapies, improving pharmacokinetic and pharmacodynamic profiles. The review highlights ligand-functionalized and stimulus-responsive nanocarriers designed for controlled drug release and targeted therapy in the liver environment. Co-modulation of NF-κB and JAK/STAT signaling via nanotechnology enhances antitumor efficacy and decreases systemic toxicity, as supported by preclinical and recent translational data. Lastly, important issues such as scalability, biosafety, and regulatory concerns are discussed. Future directions for integrating precision cancer techniques with nanomedicine are proposed. This analysis emphasizes the therapeutic potential of targeting the NF-κB and JAK/STAT pathways with nanotechnology to enhance outcomes in HCC.

**Graphical Abstract:**

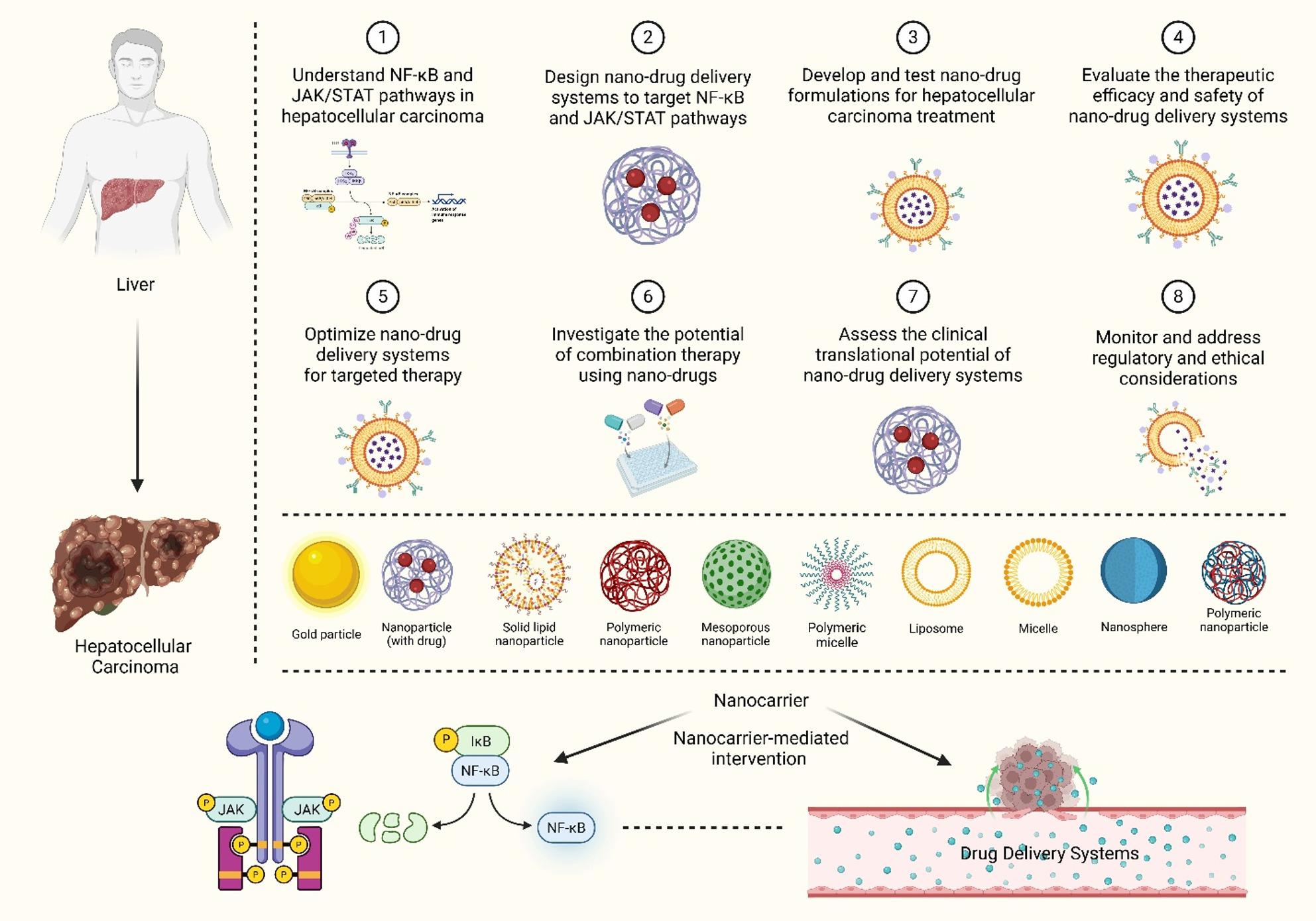

## Introduction

Hepatocellular carcinoma (HCC) was responsible for 830,180 deaths globally in 2020, making it the most prevalent primary liver cancer [1]. Additionally, HCC is a prevalent cancer often arising from liver cirrhosis, characterized by significant angiogenesis. Vascular endothelial growth factor plays a critical role in this process, with tumor-induced angiogenesis involving complex interactions. Anti-angiogenic drugs have been developed to enhance survival by inhibiting neo-angiogenesis, with sorafenib and lenvatinib as first-line treatments for advanced, incurable HCC. However, these medications come with numerous side effects [2]. The etiology of HCC is increasingly attributed to metabolic-associated fatty liver disease (MAFLD), which is prevalent in 38.77% of the global population and correlates with obesity rates. The transition of HCC causes from viral infections to metabolic disorders highlights the role of liver fibrosis and lifestyle factors such as type 2 diabetes, obesity, alcohol use, and smoking. The pathophysiological relationship between MAFLD and HCC involves oxidative stress and metabolic dysregulation, complicating early diagnosis [3]. HCC is a prevalent malignant tumor linked to chronic liver damage from hepatitis B (HBV) and C (HCV) infections, leading to inflammation, hepatocyte death, cirrhosis, and liver fibrosis. Inflammatory cell infiltration releases cytokines, notably from the interleukin-6 (IL-6) family, which significantly regulate liver processes and represent potential therapeutic targets. The study demonstrates the pathophysiology of HCC, focusing on the JAK/STAT signaling pathway activated by IL-6-type cytokines and highlights the role of mutations in genes related to the cytokine/JAK/STAT axis [4]. NF-κB plays a critical role in cancer progression. The NF-κBp65 signaling pathway shows abnormal activation in HCC tissues and Hep3B cell lines. Inhibition of NF-κB decreases the expression of invasion-related molecules and significantly reduces the proliferation and invasion of Hep3B cells. Additionally, treatment with sorafenib induced apoptosis in a dose-dependent manner, particularly when combined with NF-κB inhibition. The combination enhanced MAPK signaling suppression and reduced the anti-apoptotic protein Mcl-1. NF-κB, especially alongside sorafenib, could be an effective treatment strategy for patients with advanced-stage HCC [5]. NHE-06 exhibits anti-inflammatory properties and antitumor activity against HCC by inhibiting the NF-κB/IL-6/STAT3 inflammatory pathway. In preclinical studies, both in vitro and in HCC-bearing mice, it enhanced antitumor immunity and demonstrated therapeutic and preventative effects in a subcutaneous HCC model. The efficacy of NHE-06 depends on a functional immune response, being effective in immunocompetent mice but not in immunocompromised ones. NHE-06 may enhance antitumor immunosurveillance and could be a viable treatment for malignancies linked to inflammation [6]. COA6, a protein associated with mitochondria, plays a critical role in regulating tumor cuproptosis in HCC. Silencing COA6 inhibited malignant behavior in HepG2 and HUH7 cells, induced cuproptosis, and enhanced ROS production. An interaction between COA6 and NDUFA4L2 was observed, with COA6 depletion significantly disrupting the JAK-STAT signaling pathway. COA6 inhibits HCC progression by blocking malignancy and promoting cuproptosis [7]. Sorafenib resistance may arise through the regulation of microRNA hsa-let-7c-5p by circular RNA circ_SPECC1, affecting cell cycle proteins and the JAK-STAT signaling pathway. Furthermore, mitogen-activated protein kinase kinase 4 levels and miR-18a-z are associated with patient survival. Overall, circ_SPECC1, hsa-let-7c-5p, cell cycle, and JAK-STAT pathways could play a role in sorafenib resistance in HCC [8]. A highly cytotoxic drug, cisplatin, is used for treating various solid cancers, including liver tumors. This study evaluates the potential of gold nanoparticles (GNPs) to enhance the effectiveness of cisplatin in treating DENA-induced hepatic tumors, particularly in light of the rising incidence of hepatic tumors and the non-selective toxicity of cisplatin, while aiming to reduce renal damage. GNPs enhance cisplatin targeting to tumor sites and mitigate its renal toxicity [9]. Nanomedicines for HCC exhibit significant potential due to their stability, controlled release, and high drug loading capacity [10]. The review demonstrates targeting NF-κB and JAK/STAT pathways in hepatocellular carcinoma, with an emphasis on developing drug delivery systems using nano-drugs.

## Pathophysiology

**Fig. 1:**
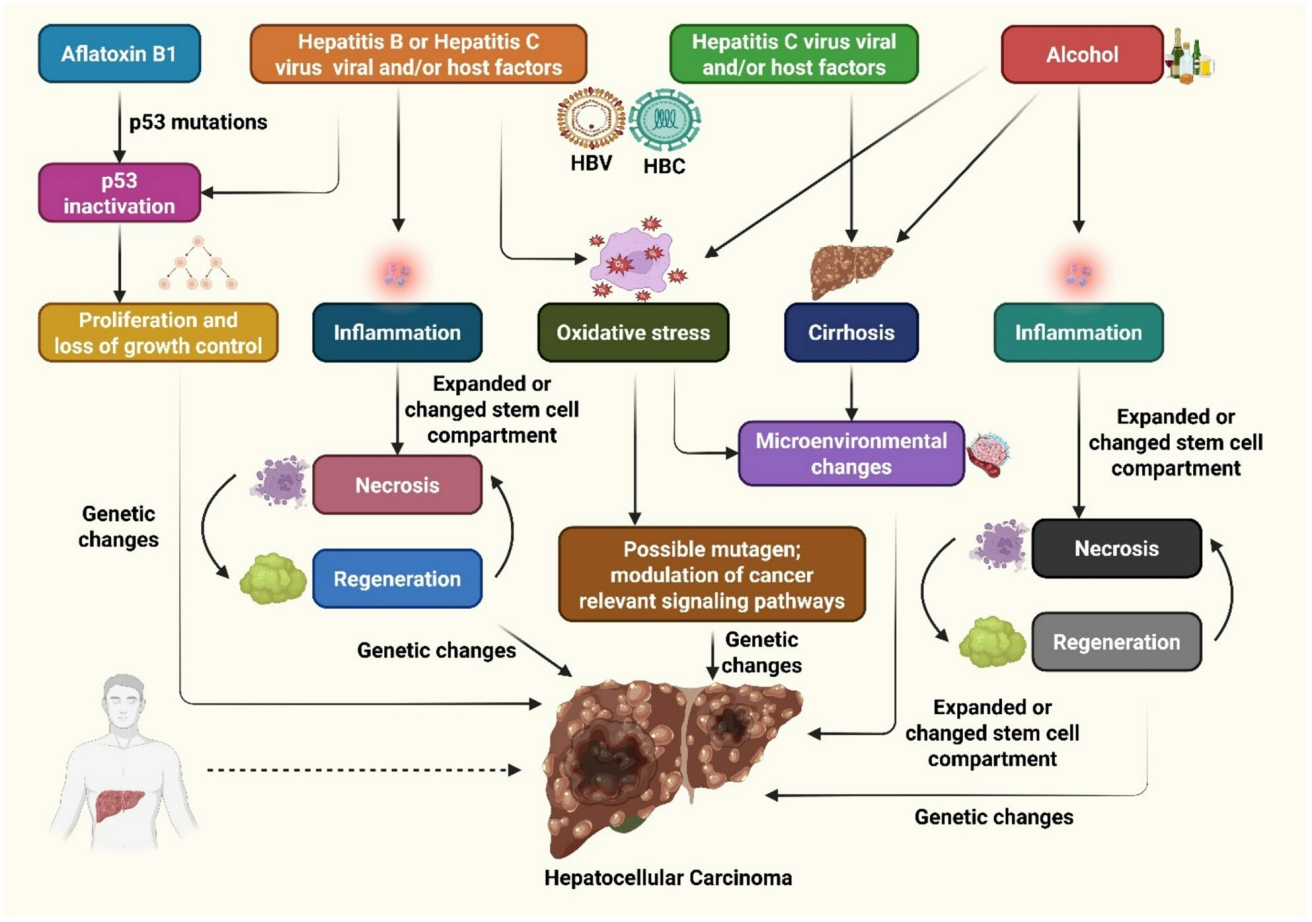
Hepatocellular carcinoma develops through multistep processes involving cirrhosis, oxidative stress, inflammation, and p53 inactivation, primarily due to alcohol intake, chronic hepatitis B and C infections, and aflatoxin B1 exposure. These factors lead to the modification of hepatic stem/progenitor cells, cycles of necrosis and regeneration, and long-term changes in the microenvironment. Ultimately, malignant transformation occurs due to accumulated genetic alterations and dysregulated signaling pathways related to cancer

The development of HCC **(**Fig. 1**)** is commonly associated with chronic liver injury leading to cirrhosis, with causes including chronic viral hepatitis (HCV and HBV), alcohol misuse, and metabolic dysfunction-related liver diseases. Approximately one-third of cirrhotic patients will develop HCC, with about 80% of cases originating from cirrhotic livers. This highlights the complex relationship between chronic liver disorders and HCC, emphasizing the need for early detection and intervention. Liver sinusoidal endothelial cells regulate fibrosis through scavenger receptors like Stabilin-1 and Stabilin-2, affecting blood composition and organ function. Stabilin deficiency may lead to increased collagen deposition and altered hepatic content in response to challenges, suggesting potential therapeutic strategies involving anti-stabilin treatments currently in clinical evaluation [11]. A study on HCC emphasizes its aggressive nature and high prevalence in individuals with liver cirrhosis. About 20% of primary liver tumors occur in non-cirrhotic livers. Variations in lipid metabolism in chronic liver disease patients are associated with HCC progression, showing notable differences in lipid content between carcinogenic and non-carcinogenic liver tissues. High cholesterol and low ceramide levels within tumors promote cell division and protect against oxidative damage. The study investigates the role of lipids in the development of HCC to improve treatment strategies, focusing on differentiating lipid changes specific to HCC from those associated with other liver diseases. Abnormal lipid metabolism in HCC patients is investigated as a potential for early detection biomarkers [12]. Individuals with HCC often share characteristics with those having end-stage renal disease (ESRD) and chronic kidney disease (CKD). Renal impairment can significantly affect the prognosis and treatment of HCC patients, complicating treatment outcomes. Causes of the comorbidity between CKD and HCC include uremia, long-term dialysis, immunosuppressants, hormonal changes, and dysbiosis. Conversely, HCC may affect renal function through hepatorenal syndrome and tumor invasion. Risk factors for both conditions include vasoactive factors, metabolic syndrome, viral hepatitis, and environmental pollutants [13]. The JAK-STAT pathway plays a critical role in lung cancer pathophysiology, affecting processes like cell differentiation and immunological escape. Both preclinical and clinical studies indicate that JAK-STAT pathway inhibitors show promise for targeted therapies in lung cancer. The JAK-STAT pathway has composition, activation, and interactions, while exploring the molecular mechanisms leading to its abnormal activation in liver cancer [14]. Liver damage and inflammation are key factors in the pathophysiology of HCC. Chronic liver inflammation leads to a fibrotic reaction and ultimately cirrhosis. The tumor microenvironment (TME) plays a significant role in disease progression [15]. HCC can be managed locally with radiofrequency ablation (RFA). The superiority of surgical excision over RFA remains debated for patients with up to three tumors ≤ 3 cm. HCC frequently recurs post-RFA because of incomplete ablation, with studies revealing molecular alterations in tumor cells. Key factors include EMT and cancer stemness, influenced by microRNA-induced hypoxia-inducible factor-1 [16]. Autophagy is an internal lysosomal destruction process that cells utilize to maintain energy balance, significantly influencing liver diseases, including hepatitis virus infection, alcoholic liver disease, nonalcoholic fatty liver disease, liver cirrhosis, and HCC. Increased autophagy influences liver pathophysiology through immune response modulation, while impaired autophagy may lead to HCC. Regulating autophagy demonstrated a targeted treatment strategy for human liver cancer [17]. Numerous physiological and biochemical events contribute to HCC progression, primarily influenced by viral hepatitis and alcoholic cirrhosis. The transcription factor Twist is implicated in HCC, facilitating EMT and leading to biochemical changes such as increased cell division, reduced apoptosis, and deregulated cell cycle. These changes lead to hepatocellular metastasis, angiogenesis, and cellular migration alterations [18].

## NF-κB pathways

**Fig. 2:**
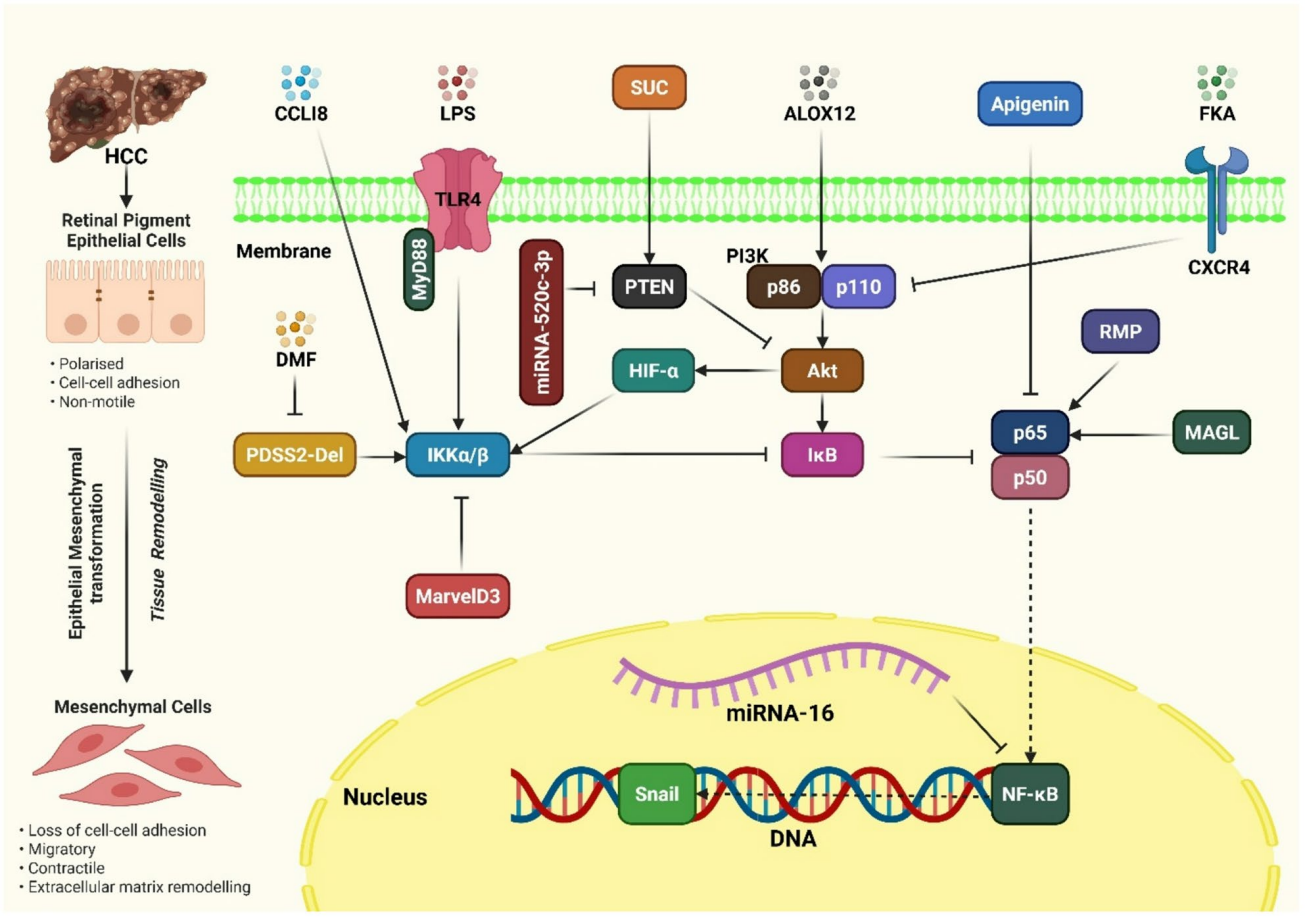
It illustrates the signaling pathways in hepatocellular carcinoma (HCC) showing how PI3K/Akt and NF-κB signaling, activated by external stimuli like CCL18 and LPS, involve receptors such as TLR4 and CXCR4. Crosstalk with HIF-α and microRNAs promotes epithelial-mesenchymal transition (EMT), tissue remodeling, and mesenchymal traits via transcriptional regulators like Snail. Natural substances such as apigenin and DMF inhibit NF-κB activation

The primary causes of death in chronic liver disease are HCC and hepatic cirrhosis. Chronic liver injury triggers inflammatory responses that promote hepatic fibrosis and HCC. The study investigates the role of the transcription factor NF-κB in hepatocellular damage, liver fibrosis, and HCC. NF-κB is a potential target for the prevention or treatment of liver fibrosis and HCC, though excessive inhibition may threaten hepatocyte viability. Therefore, it is essential to identify therapeutic strategies that can modulate NF-κB activity safely or target nonparenchymal cells specifically [19]. Chemoresistance in HCC is linked to aberrant NF-κB pathway activation, with microRNAs like miR-26b potentially serving as new anti-cancer targets. Manipulating miR-26b expression affects NF-κB signaling in HCC cell lines, with restoration of miR-26b leading to decreased NF-κB activity and increased sensitivity to doxorubicin treatment [20]. A study investigated the role of NF-κB in HCC by profiling the activation of 45 transcription factors in the HEP3B cell line. A luciferase-based screening assay was developed to identify potential drugs that target NF-κB. The subset of HCCs and precancerous cirrhosis tissues exhibits active NF-κB signaling, indicating that NF-κB inhibitors like ornithogalum are potential preventive and targeted therapies for these conditions [21]. Another study investigated the effects of inhibiting NF-κB activity alongside doxorubicin treatment to evaluate the roles of NF-κB and IκBα in HCC SMMC7721 cells. SMMC7721 cells exhibit higher expression of NF-κB subunits (P50 and P65) and lower levels of cytosolic IκBα compared to normal liver cells. NF-κB overexpression and IκBα downregulation promote HCC cell proliferation, while mIkBα expression can limit growth and NF-κB translocation, enhancing the effectiveness of doxorubicin treatment [22]. Furthermore, a study found that the role of CXCL5 in HCC was explored, revealing its involvement in preventing cell survival and metastasis. CXCL5 levels were analyzed through RT-qPCR and western blot, while functional assays assessed invasion and proliferation. Increased CXCL5 expression correlated with lower overall survival in HCC patients [23]. The current HCC gene signature consists of 216 genes within these DTGs. A limited overlap (24–46 genes) exists between NF-κB target genes in other cell lines, indicating specificity to HCC. Functional annotation shows these DTGs are involved in various NF-κB-related processes, including immune response and signaling pathways. Notably, 82 DTGs encode secretory proteins, such as DKK1 and CCL2, linked to HCC [24]. HCC is heavily modulated by NF-κB, particularly through the action of β-arrestin1 (ARRB1), which enhances NF-κBp65 activity. ARRB1 promotes both hepatocellular carcinogenesis and HCC progression. Studies measured NF-κBp65 and its phosphorylated form (p-p65) in paracancerous tissues, primary HCC, and normal liver. Results showed significantly elevated p-p65 levels in models of hepatic inflammation and in patients with inflammation-related HCC. p65-deficient mice exhibited reduced HCC incidence and tumor size, indicating inhibited tumor cell growth. ARRB1 enhances p65 phosphorylation via GSK3β/mTOR signaling, which contributes to malignant cell proliferation. NF-κBp65 phosphorylation, mediated by ARRB1 in inflammatory contexts, is a key driver of HCC and suggests this mechanism as a potential therapeutic target for HCC [25]. In a study of human HCC tissues from eight patients, the expression of the NF-κB monomer p65 was analyzed in comparison to surrounding tissues. It explored the effects of NF-κB suppression in oxidatively stressed HuH7 HCC cells. The overexpression of p65 was noted in six patients, and mIκBα introduction reduced NF-κB activation and translocation. This suppression increased HuH7 cell sensitivity to H2O2-induced growth inhibition and enhanced caspase-3 activity, suggesting that mIκBα-mediated suppression of NF-κB promotes OS-induced cell death in HCC cells [26]. Hepatitis B virus HCC (HBV-HCC) is demonstrated to be primarily regulated by microRNAs. The miR-98-5p inhibits HBV production and the aggressive behaviors of HBV-HCC cells by targeting NIK [27]. Non-coding microRNAs (miRNAs) like miR-342-3p influence biological functions in cancer cells, particularly in HCC. Overexpression of miR-342-3p was validated through qRT-PCR, and the MTT assay demonstrated a significant reduction in HCC cell proliferation. miR-342-3p targets the NF-κB pathway, with identified target genes including IKK-γ, TAB2, and TAB3. qRT-PCR results indicated that miR-342-3p downregulates NF-κB pathway downstream genes while its inhibition leads to their upregulation. Furthermore, restoring HCC proliferation through overexpressing IKK-γ, TAB2, and TAB3 indicated their role in miR-342-3p’s effects. Analysis of HCC samples showed downregulation of miR-342-3p [28]. A study assessed HCC cell changes in morphology, migration, invasion, and signaling pathways with or without inflammatory cytokines. High E-cadherin expression was linked to better recurrence-free survival, while increased p65 and Snail expression correlated with poorer outcomes. The study found a signaling axis in the inflammatory tumor microenvironment promoting HCC recurrence, suggesting therapies that reduce hepatic inflammation or target NF-κB-mediated Snail expression may prevent recurrences after hepatectomy [29]. One key mechanism contributing to growth, distant metastases, and treatment resistance in HCC is EMT. Targeting EMT provides a promising approach for HCC immunotherapy, with the NF-κB pathway identified as a critical regulatory mechanism (Fig. 2). Immunotherapy influences EMT regulation in HCC cells [30]. Periplocin demonstrates potential in inhibiting HCC based on cytotoxicity tests and murine models. It reduced cell viability in human HCC cells. Periplocin is developed as an effective treatment for HCC through mechanisms involving apoptosis, cell cycle arrest, and MDSC formation inhibition [31]. PPARα ligands inhibit cancer growth, though their role in hepatocarcinogenesis is unclear. In a study, PPARα-knockout (PPARα-/-) mice showed increased susceptibility to diethylnitrosamine (DEN)-induced HCC (80% vs. 43%, and fewer apoptotic cells in HCCs compared to wild-type mice (*P* < 0.01). Enhanced cell proliferation was observed in PPARα-/- mice, indicated by Ki-67 staining and changes in cyclin-D1 and p15 levels. PPARα expression in HCC cells inhibited division and induced apoptosis, linked to decreased NF-κB activity and direct binding to the IkBα promoter [32]. Moreover, the combination therapy using sorafenib and radiation therapy may improve treatment outcomes. Sorafenib inhibits NF-κB expression, which is associated with resistance to therapies [33]. New surgical specimens from 144 HCC patients were analyzed to assess Pin1 and NF-κB-p65 expression, along with measuring NF-κB activation through EMSA. HCC exhibited higher Pin1 levels compared to the surrounding liver tissue. Additionally, phosphorylated NF-κB-p65 was associated with Pin1 levels, promoting NF-κB activation. This activation led to increased angiogenesis, accelerated cell cycle progression, and inhibition of apoptosis. Conversely, Pin1 knockdown resulted in decreased NF-κB activation and reduced tumor cell growth. Pin1 inhibitors also shortened NF-κB activation and cell proliferation in HCC [34]. Another study investigates whether aspirin can improve HCC prognosis by targeting the collagen-synthesizing enzyme P4HA2. It influences P4HA2 expression and its anti-fibrotic effects. It also inhibited liver tumor formation in mouse models by reducing collagen deposition, and higher P4HA2 levels in patients correlated with worse survival outcomes. NF-κB activates P4HA2 transcription, and LMCD1-AS1 enhances P4HA2 expression by sponging let-7 g [35]. Radioresistant HCC cell lines exhibited increased Aurora-A levels compared to parental cells. Overexpression of Aurora-A reduced radiosensitivity by lowering apoptosis, while its knockdown heightened radiosensitivity by promoting apoptosis. Aurora-A also augmented NF-κB signaling through increased IκBα protein expression and activity, leading to the upregulation of apoptotic regulators like Mcl-1 and Bcl-2. Targeting Aurora-A may serve as an effective strategy for enhancing radiotherapy efficacy in HCC [36]. In this study, the interaction between sorafenib and Toll-like receptor 3 (TLR3) in HCC was explored. TLR3 activation could bolster sorafenib’s anticancer efficacy in HCC, presenting a potential new treatment strategy when used in combination with TLR3 activators [37].

## JAK/STAT pathways

**Fig. 3:**
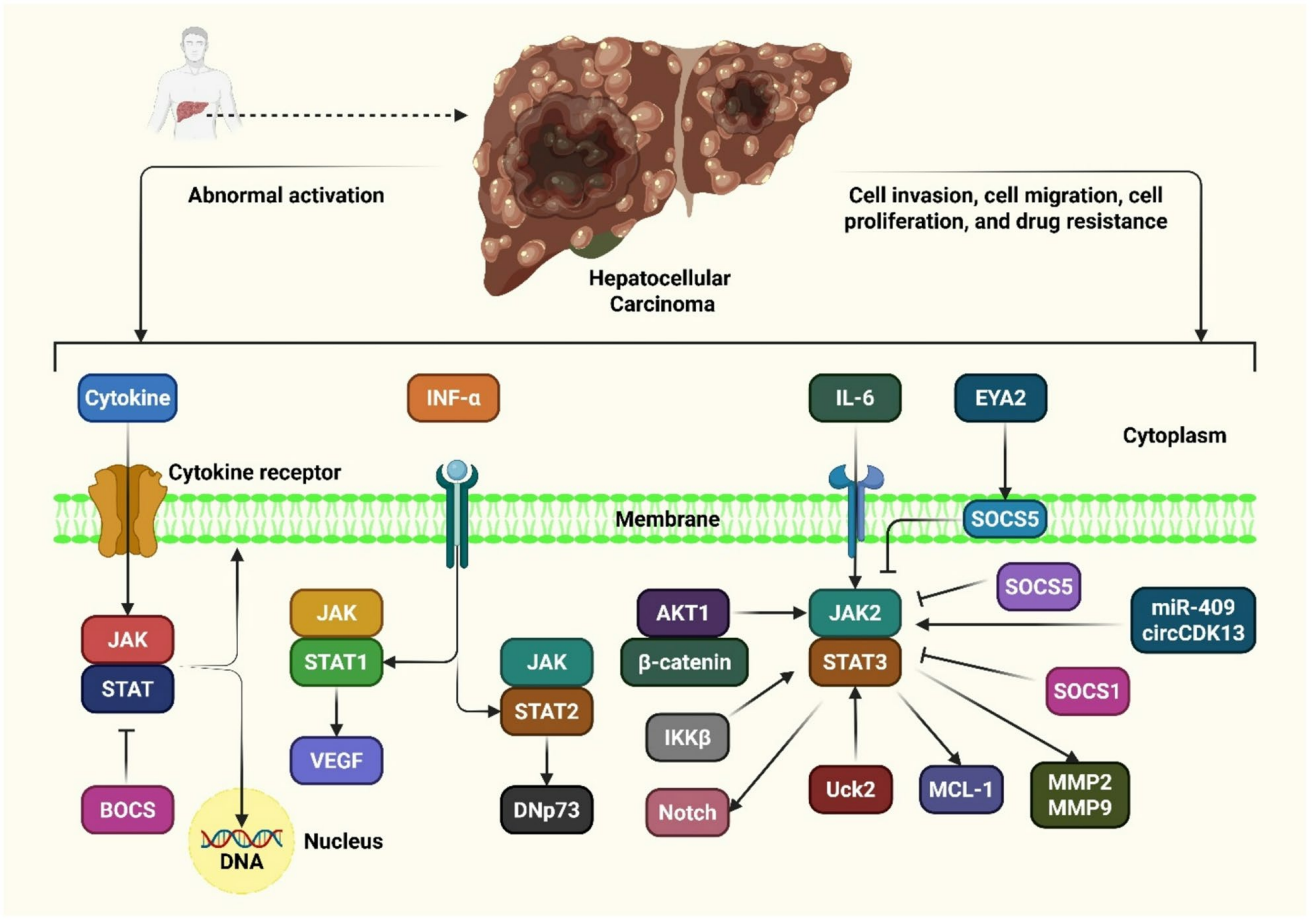
The representation of liver cancer reveals that cytokines such as IL-6 and IFN-α activate JAK/STAT signaling. This activation leads to nuclear transcription of genes associated with angiogenesis, proliferation, invasion, migration, and drug resistance. Additionally, signaling is amplified through interactions with pathways including AKT/β-catenin, NF-κB, Notch, and non-coding RNAs (miRNAs, circRNAs). Negative regulators like SOCS proteins are often dysregulated in cancer, impacting pathway functionality

HCC involves complex interactions in its development and tumorigenicity. Several IL-6/STAT3 inhibitors, such as S31-201, IL-6 mAb, Madindoline A, C188-9, and curcumin, have been developed, underscoring the importance of this pathway in liver cancer therapies [38]. In this study, AKT1 and β-catenin oncogenes were used to establish an HCC tumor model, followed by the identification of cancer cells with stemness properties through flow cytometry. Analysis revealed that 14.4% of HCC patients had co-activated Akt/mTOR and Wnt/β-catenin pathways, correlating with reduced survival rates. The presence of a tumor cell fraction exhibiting stem/progenitor traits may contribute to treatment resistance. Jak/Stat inhibitors showed promise in inhibiting tumor growth in Akt/β-catenin-driven HCC, suggesting that targeting the JAK/STAT pathway could be a viable strategy to address drug resistance in these tumors [39]. Both healthy and cancerous cells utilize the JAK/STAT signaling system for crucial biological functions [40]. A study identifies the JAK and STAT pathway as a crucial signaling cascade in HCC pathophysiology. The chronic activation of JAK/STAT signaling **(**Fig. 3**)** leads to oncogenic mechanisms like immunological escape, evasion of apoptosis, and unchecked cell proliferation during hepatocarcinogenesis. Therapeutic developments targeting the JAK/STAT pathway for treating HCC are discussed, with a focus on preclinical and clinical research involving modulators and inhibitors [41]. Research indicates that individuals with HCC often experience iron overload, associated with poor prognosis and lower survival rates. Iron metabolism dysregulation in HCC involves several proteins and pathways, particularly the JAK/STAT system. Additionally, targeting HCC with JAK/STAT pathway inhibitors requires further investigation regarding their impact on hepatic iron metabolism [42]. Tumor models from patient-derived xenografts are valuable for studying the biology of diseases and discovering new drugs. Whole-exome sequencing of over 60 HCC PDX models revealed mutations in the JAK1 gene in four models. These mutations were investigated for their transforming potential in cell lines, showing that the JAK1S703I mutation may enhance cell proliferation in vitro. The JAK1S703I mutant patient-derived xenografts model was sensitive to ruxolitinib, a JAK1/2 inhibitor, while other models showed no response. Treatment with ruxolitinib significantly inhibited STAT3 phosphorylation in tumor tissues [43]. A study assessed cell proliferation, apoptosis, and tumor progression. Knockdown of these miRs led to apoptosis and reduced cell proliferation, while targeting SOCS2. Activity of the JAK/STAT pathway was inversely related to SOCS2 expression, and knockdown of miR-196a or miR-196b inhibited xenograft tumor growth [44]. Another study investigates the Ras and Jak/Stat signaling pathways in human HCC. Abnormal methylation in specific genes indicated a potential link to poor survival outcomes. The apoptosis-inducing effects of combining demethylating agents like Zebularine with pathway inhibitors suggest a therapeutic way for liver cancer treatment [45]. Furthermore, a study explores the clinical relevance of key genes in the JAK-STAT pathway for HCC. It found 370 patients with HCC, and GSE14520, consisting of 212 hepatitis B virus-infected patients, to evaluate the diagnostic and prognostic significance using joint-effect analyses. Molecular mechanisms indicated STAT6’s involvement in lipid metabolism and cell cycle processes. Genes may serve as valuable biomarkers for diagnosing and predicting the prognosis of HCC [46]. Oncogenesis and tumor progression are promoted by the EphB4 tyrosine kinase receptor, and its inhibition is a potential strategy for solid tumor treatment. This study evaluates cantharidin, a novel EphB4 inhibitor, in HCC therapy. Elevated EphB4 levels were found in HCC patient samples, correlating positively with p-JAK2 levels. Cantharidin’s efficacy in reducing viability in HCC cell lines was positively associated with EphB4 levels. It demonstrated significant antiproliferative effects on tumor xenografts. Cantharidin was shown to bind to EphB4, inhibiting it at the mRNA and protein levels. The absence of EphB4 in specific cell lines indicated that EphB4 knockdown reduced cantharidin’s effects, while overexpression exacerbated them. It induces intrinsic apoptosis by inhibiting both PI3K/Akt and novel JAK2/STAT3 pathways via EphB4 targeting [47]. After a viral infection, the pathways associated with IFN gamma trigger mitochondrial dysfunction, particularly involving Jak family proteins. Momelotinib, a PARP1-targeting drug, could inhibit HCC progression. The research assessed the synergistic effects of momelotinib and sorafenib on liver cancer linked to hepatitis. Results indicated increased Jak1 and Jak2 expression in vHCC compared to normal tissues, with momelotinib reducing Jak2 levels and HCC cell proliferation. It effectively inhibited the IFNGR-JAK-STAT pathway by downregulating key transcription factors and biomarkers. The combination of momelotinib and sorafenib effectively reduced vHCC growth and tumor burden. Momelotinib enhances sorafenib’s therapeutic efficacy by downregulating pro-cancer pathways and promoting apoptotic gene expression in vHCC cells [48]. Investigating the role of circularRNA-9119 (circ9119) in HCC cells revealed increased expression of circ9119 and JAK1. Moreover, miR-26a was found to target the JAK1 3′-UTR, leading to decreased JAK1 levels upon miR-26a mimic transfection. The JAK1 inhibitor baricitinib similarly influenced cell growth and death patterns as circ9119 silencing. Thus, circ9119 can regulate HCC cell proliferation and apoptosis through competitive binding with miR-26a, targeting the JAK1-STAT3 pathway, presenting a novel understanding of circ9119’s influence in HCC [49]. All four human HCC cell lines exhibited an anti-proliferative response to IFN-α, with PLC/PRF/5 showing the strongest reaction due to its high IFN receptor expression. IFN-α inhibits proliferation via the MAPK pathway by decreasing cell growth and delaying the G1/S transition, without inducing apoptosis [50]. Analysis of human HCC tissues revealed higher EPHX1 protein levels compared to non-tumor tissues. Experimental approaches, including cell line manipulation and xenograft models, indicated that EPHX1 suppresses regorafenib’s effects on cell division and apoptosis, while its knockdown enhanced drug efficacy. Digital gene expression sequencing demonstrated EPHX1’s involvement in resistance mechanisms, revealing that inhibiting the JAK/STAT pathway with HY-N1447 decreased EPHX1-mediated resistance. Thus, EPHX1 is identified as a significant resistance-related gene in advanced HCC with implications for regorafenib treatment [51]. Therapeutic options for HCC are limited, with the JAK/STAT signaling pathway playing a significant role in its carcinogenesis. Novartis Pharmaceuticals has introduced Ruxolitinib (INC424), a JAK inhibitor showing promise in clinical trials for myelofibrosis. Ruxolitinib inhibits JAK/STAT signaling in HCC cells by reducing pSTAT1 and pSTAT3 expression, leading to decreased cell proliferation and colony formation [52]. Src homology domain 2 containing tyrosine phosphatase-2 (SHP-2) inhibits the immunoregulatory and antiproliferative effects of type I IFN-α/β. Inhibition of SHP-2 by small molecules, such as quercetin, may enhance the effectiveness of type I IFNs. Quercetin was identified as a potent SHP-2 inhibitor and, through computational modeling, was shown to favorably interact within the SHP-2 phosphatase domain [53]. Additionally, EYA2 deletion in liver cells accelerated HCC development, and aberrant methylation also contributed to reduced EYA2 expression. Mechanistically, EYA2, along with DACH1, inhibits HCC progression by regulating SOCS3 and blocking the JAK/STAT signaling pathway [54]. HCCs show overexpression of microRNA-761 (miR-761), whose inhibition affects mitochondrial function and limits HCC spread. In six cell lines, exosomal miR-761 was observed. Methodologies included transwell migration tests and CCK-8 assays to assess the role of miR-761 in HCC cells, alongside luciferase reporter experiments for identifying target genes in normal fibroblasts (NFs). The study explored the JAK2/STAT3 signaling pathway in cancer-associated fibroblasts using inhibitors AZD1480 and C188-9. The miR-761 alters the tumor microenvironment by stimulating NFs via the JAK2/STAT3 pathway and suppressor of cytokine signaling 2 [55]. Moreover, a study investigates the role of artesunate in HCC, focusing on its impact on the JAK-STAT signaling pathway activated by IL-6. In an experimental model using nitrosodiethylamine, rats exhibited increased liver pathophysiological markers and tumor biomarker levels, indicating liver tumorigenesis. Artesunate treatment (25 mg/kg) reduced these markers and showed anti-tumor and anti-proliferative effects. Immunoblot analysis indicated that nitrosodiethylamine elevated JAK-STAT pathway components and anti-apoptotic markers, whereas artesunate countered these effects, demonstrating its potential to inhibit JAK-STAT signaling and enhance apoptosis in nitrosodiethylamine-induced HCC [56].

## Crosstalk Between NF-κB and JAK/STAT signaling in hepatocarcinogenesis and drug resistance

Inflammatory cell infiltration and ongoing hepatocyte loss are critical aspects of HCC’s pathophysiology. The IKKβ-dependent NF-κB pathway is crucial for the survival of hepatocytes and the regulation of growth factors and cytokines in liver inflammation, particularly IL-6, which acts via the transcription factor STAT3. The study focuses on the roles of STAT3 and NF-κB in liver cancer and explores their potential as therapeutic targets [57]. A cycle of necro-inflammation and hepatocyte regeneration from chronic liver damage causes genetic abnormalities in hepatocytes, resulting in initiated cells and ultimately leading to HCC. NF-κB, signal transducer and activator of transcription, and stress-activated MAPK pathways are significant participants in various processes, demonstrating potential therapeutic targets and complex interactions among these pathways [58]. In human cancer cells, the expression and phosphorylation of STAT3 increase as HCC progresses. STAT3, triggered by growth factors and cytokines, regulates cancer proliferation by inhibiting apoptosis and promoting EMT and autophagy. Its overexpression is associated with chemoresistance and radioresistance in HCC. The oncogenic role of STAT3 is significant in influencing carcinogenesis and therapy resistance. It impacts the response to anticancer drugs, particularly concerning non-protein-coding transcripts that regulate STAT3 signaling [59]. A study investigates the mechanisms and consequences of long-term indomethacin use in HCC. Indomethacin promotes tumor recurrence, intrahepatic metastasis, and distant metastasis in HepA mice models. Long-term indomethacin also elevates expression of PD-1 and PD-L2 in the programmed death-1 pathway. The reduced production of TNF-α and IFN-γ in γδ T cells due to indomethacin can be reversed by blocking PD-1 and PD-L2. Moreover, it activates the JAK/STAT3 and TRIF/NF-κB pathways, enhancing PD-1 and PD-L2 expression in γδ T cells. Overall, long-term indomethacin use is associated with poor prognoses in HCC by inhibiting TNF-α and IFN-γ and increasing PD-1 and PD-L2 expression through the TRIF/NF-κB and JAK/STAT3 pathways [60]. IKKβ inhibits early liver carcinogenesis by preventing hepatocyte apoptosis and compensatory proliferation. The study of the role of IKKβ in late tumor promotion demonstrated that its deletion increases tumor-initiating cells and accelerates HCC development due to elevated ROS, activating JNK and STAT3. Hepatocyte-specific STAT3 ablation suppressed HCC development and demonstrated the significant negative crosstalk between NF-κB and STAT3 in regulating liver cancer onset and progression [61]. In sorafenib-acquired resistant tissue, 827 mRNAs were expressed differently compared to controls. Functional enrichment analysis revealed dysregulated mRNAs associated with drug-resistant signaling pathways, including MAPK, JAK-STAT, TGF-β, and cytochrome P450 metabolism. CDK1, CDKN1A, and TAPBP might be prognostic indicators of sorafenib resistance in HCC. Networks of transcription factor (TF)-mRNA interactions identified 18 TFs that regulated differentially expressed mRNAs. MYC was anticipated as a predictive TF for drug resistance, while NFKB1 emerged as a key node in the TF-mRNA networks. mRNAs and transcription factors are important biomarkers for identifying therapeutic targets in sorafenib-resistant HCC [62]. The enzyme AKR1C3, part of the aldo-ketoreductase family, is crucial for the growth and metastasis of HCC. AKR1C3 levels are significantly elevated in HCC and correlate with reduced survival rates. It regulates HCC cell growth and metastasis through NF-κB signaling, inducing proinflammatory molecules that activate the IL6/STAT3 pathway, thereby enhancing tumor cell proliferation and invasion. A feedback loop exists where STAT3 promotes AKR1C3 transcription. Inhibitors of AKR1C3, like Indocin and medroxyprogesterone acetate, may inhibit tumor growth and induce apoptosis in HCC cells, suggesting targeting the AKR1C3/NF-κB/STAT3 signaling loop as a potential therapeutic strategy [63]. CRP-related signaling pathways, including NF-κB and IL-6-STAT3, were identified through transcriptome sequencing and GSEA. CRP overexpression enhances HBV replication by increasing cccDNA, pgRNA, and viral protein synthesis, while promoting cell migration and proliferation and decreasing apoptosis. Silencing CRP led to decreased tumor growth, HBV replication, and viral protein levels in both cellular and animal models. Transcriptome analysis shows that CRP influences additional carcinogenic processes, including OS and JAK-STAT signaling. CRP knockdown led to an increase in anti-apoptotic proteins like BCL-2 and a decrease in inflammatory cytokines such as TNF-α and IL-6. CRP plays a significant role in the development of HBV-driven HCC by modulating inflammatory pathways, indicating its potential as a therapeutic target to inhibit HBV replication and HCC progression [64].

## Nano-drug targeting NF-κB pathway

HCC is a leading cause of liver cancer and a significant cause of cancer mortality worldwide. It highlights the limitations of conventional chemotherapy for advanced HCC and introduces sorafenib, an oral kinase inhibitor that induces cancer cell apoptosis, inhibits tumor growth, and improves survival chances, despite its poor solubility and low bioavailability. The use of NPs in enhancing drug targeting and therapeutic efficacy for HCC has gained attention, with various types such as polymer, lipid, silica, and metal NPs developed to improve sorafenib’s effectiveness [65]. Portulacerebroside A (PCA) has shown suppressive effects on HCC cells. This study investigates the molecular mechanisms of PCA’s action and the efficacy of trimethyl chitosan–cysteine (TMC-Cys) nanocarriers in enhancing PCA’s delivery for HCC treatment. TMC-Cys significantly improved PCA’s performance by inhibiting HCC cell migration, proliferation, and invasiveness, reducing tumorigenesis in immunocompetent mice, and preventing metastases in xenograft models. Bioinformatics analyses identified TLR4 and EGFR as key targets of PCA involved in the immune checkpoint pathway. PCA increased T cell activity in HCC models by downregulating TLR4 and EGFR levels, although activation of these receptors reversed the effects. The findings indicate PCA’s immunomodulatory potential alongside its tumor-suppressive properties, with TMC-Cys nanocarriers enhancing PCA’s therapeutic efficacy [66]. Akt is essential for apoptosis and inflammation in liver cancer development. This study explored the Akt-associated signaling pathway involving NF-κB and Bad using Akt-knockout mice and liver cancer cells. Carnosic acid was found to significantly slow liver cancer progression. Akt expression was higher in cancer cell lines compared to normal cells. Akt-knockout cancer lines affected apoptosis and inflammation by inhibiting NF-κB activation and inducing apoptosis. Carnosic acid NPs, mimicking Akt-knockout effects, may mitigate inflammation and enhance apoptosis by modifying NF-κB activity and activating caspase-3 through the Bad pathway. Carnosic acid plays a crucial role in mitigating inflammation and promoting apoptosis through the Akt signaling pathway, which is significantly linked to the progression of liver cancer [67]. A study developed a zinc oxide-superparamagnetic iron oxide-silver nanocomposite using the mycelia of the fungus *Fusarium oxysporum*, which was loaded with the cancer drug sorafenib (Nexavar). Characterization techniques such as FTIR, SEM, TEM, and UV-visible spectroscopy were used. Sorafenib is the only systemic treatment for HCC, but its effectiveness is inhibited by side effects such as toxicity, nausea, and alopecia. The study focused on improving the anticancer effects of sorafenib by using a synthesized nanocomposite as a drug carrier, after a month of treatment with diethyl nitrosamine and carbon tetrachloride in male albino rats [68]. GC-triptolide-NPs demonstrate excellent drug loading and reduced toxicity compared to free triptolide. These provide prolonged release and high liver tumor accumulation, enhancing cellular absorption in vitro via the asialoglycoprotein receptor. In vivo, GC-triptolide-NPs effectively lower tumor sizes while preserving triptolide’s pro-apoptotic and anti-proliferative properties. These also inhibit TNF/NF-κB/BCL2 signaling, leading to cancer cell death, thus presenting a promising strategy for minimizing systemic toxicity and controlling liver cancer progression [69]. In this study, the use of NF-kB signal pathway inhibitors was investigated to reduce the phagocytosis of folic acid-conjugated pullulan acetate (FPA/EPI) NPs loaded with epirubicin (EPI) by Kupffer cells (KCs). The NPs were formed through dialysis, and various inhibitors were used to preincubate rat KCs. Fluorometry assessed the effect of these inhibitors on NP absorption, while bead-based multiplex flow cytometry measured cytokine levels of TNF-α, IL-1β, and IL-6. Significant time-dependent inhibitory effects were observed for NY, AMR, and Col, with PDTC + NY showing the highest inhibitory effect (14.62% uptake rate). Inhibitors significantly modified proinflammatory cytokine levels, particularly reducing TNF-α. The NF-κB pathway is vital, and NP entry into KCs primarily occurs through clathrin- and caveolae-mediated endocytosis. This inhibition approach could improve treatment efficacy and reduce toxicity in cancer nanodrug delivery systems [70]. Regorafenib reduced tumor growth and decreased proteins associated with tumor progression, including phosphorylated ERK and NF-κB p65. Both intrinsic and extrinsic apoptotic pathways were activated by treatment, while liver shape and body weight remained unaffected. Regorafenib effectively inhibits tumor growth in SK-Hep1/luc2 and Hep3B 2.1-7 tumor-bearing mice by suppressing ERK/NF-κB activation [71]. A study developed chitosan NPs for the co-delivery of the anticancer drug 5-fluorouracil (5-Fu) and aspirin, aiming to enhance the synergistic antitumor effects while addressing the inflammation-cancer connection. The combination of aspirin and 5-Fu via chitosan NPs notably increased drug concentration within the cells, leading to greater growth inhibition and apoptosis induction through COX-2 suppression and NF-κB inhibition [72]. Another study explores the role of GLPS in modulating macrophage polarization in HCC. GLPS treatment slowed the development of Hepa1-6 allografts and increased the expression of the M1 marker CD86 while decreasing M2 markers such as CD206 and Arg-1, along with inflammation-related cytokines. GLPS enhances macrophage phagocytic activity and NO production, indicating a change towards M1 polarization. Mechanistically, GLPS activated the MAPK/NF-κB signaling pathway, enhancing phosphorylation of proteins involved in M1 activation. This position GLPS as a novel therapeutic agent for HCC through the control of macrophage polarization [73]. Ferroptosis in liver cancer cells is influenced by aspirin, which, in treated HepG2 cells, increased expression of four ferroptosis-related drivers while decreasing five suppressors. Aspirin inhibits HCC growth via suppression of NF-κB-activated SLC7A11 transcription, thereby promoting ferroptosis. Combining aspirin with ferroptosis inducers may present a promising therapeutic strategy for HCC [74]. Furthermore, a study demonstrates the development of rutin poly(lactic co-glycolic acid) (PLGA) NPs intended for HCC treatment in rats. Utilizing a double emulsion evaporation method and using a Box-Behnken design for optimization, the research assessed various parameters, including size, drug-loading capacity, and stability. Results indicated significant improvements in hepatic, hematologic, and renal biochemical markers during preclinical evaluations of rutin-PLGA-NPs administered orally. Enhanced activity levels were noted in antioxidant enzymes and inflammatory mediators impacting liver tissue. Rutin-PLGA-NP treatment led to reduced proinflammatory cytokines and a lower incidence of hepatic nodules, necrosis, and other inflammatory conditions [75]. Arsenic trioxide effectively inhibits angiogenesis and metastasis in HCC, particularly in multidrug-resistant HCC cells, which are more susceptible to arsenic trioxide. Experiments utilizing multidrug-resistant-HCC cells involved various techniques, including transfection and immunoprecipitation. Multidrug-resistant-HCC cells adapt to OS, while arsenic trioxide disrupts this process by targeting the 14-3-3η/NF-κB feedback loop. Increased ROS in multidrug-resistant cells activates NF-κB signaling, enhancing 14-3-3η transcription, which in turn sustains NF-κB activation. Arsenic trioxide inhibits 14-3-3η production at the mRNA level and directly promotes its degradation, thereby reducing drug resistance. Ultimately, the 14-3-3η/NF-κB feedback loop is essential in maintaining the multidrug-resistant phenotype in HCC [76]. HCC treatment was advanced using dual active tumor targeting alongside synergistic medication through protein shell-oily core nanocapsules. The approach involved co-delivering sorafenib as a phospholipid complex and the herbal quercetin. Lactoferrin shell was applied to target lactoferrin receptors on HCC cells, while lactobionic acid and glycyrrhetinic acid aided dual targeting. The resulting dual-targeted lactobionic acid /lactoferrin-nanocapsules showed enhanced internalization in HepG2 cells and increased anticancer efficacy in vivo, significantly downregulating pro-inflammatory mRNA levels and reducing liver damage in diethylnitrosamine-induced HCC mice. This research indicates the potential of dual-targeted lactoferrin-nanocapsules for combined sorafenib and quercetin therapy against HCC [77]. Moreover, a study explores the application of carbon-based materials, specifically triptycene-derived three-dimensional nanographene NPs, in targeting human hepatocellular cancer cells (HepG2). It demonstrates that nanographene NPs selectively accumulate in HepG2 cells, inducing ROS that trigger apoptosis, while sparing normal liver cells (HL7702). The apoptosis in HepG2 cells is induced by ROS-mediated mechanisms and the activation of the IKK/NF-κB signaling pathway, demonstrating insights for developing advanced chemotherapy treatments to kill cancer cells [78].

## Nano-drug targeting JAK/STAT pathway

**Fig. 4:**
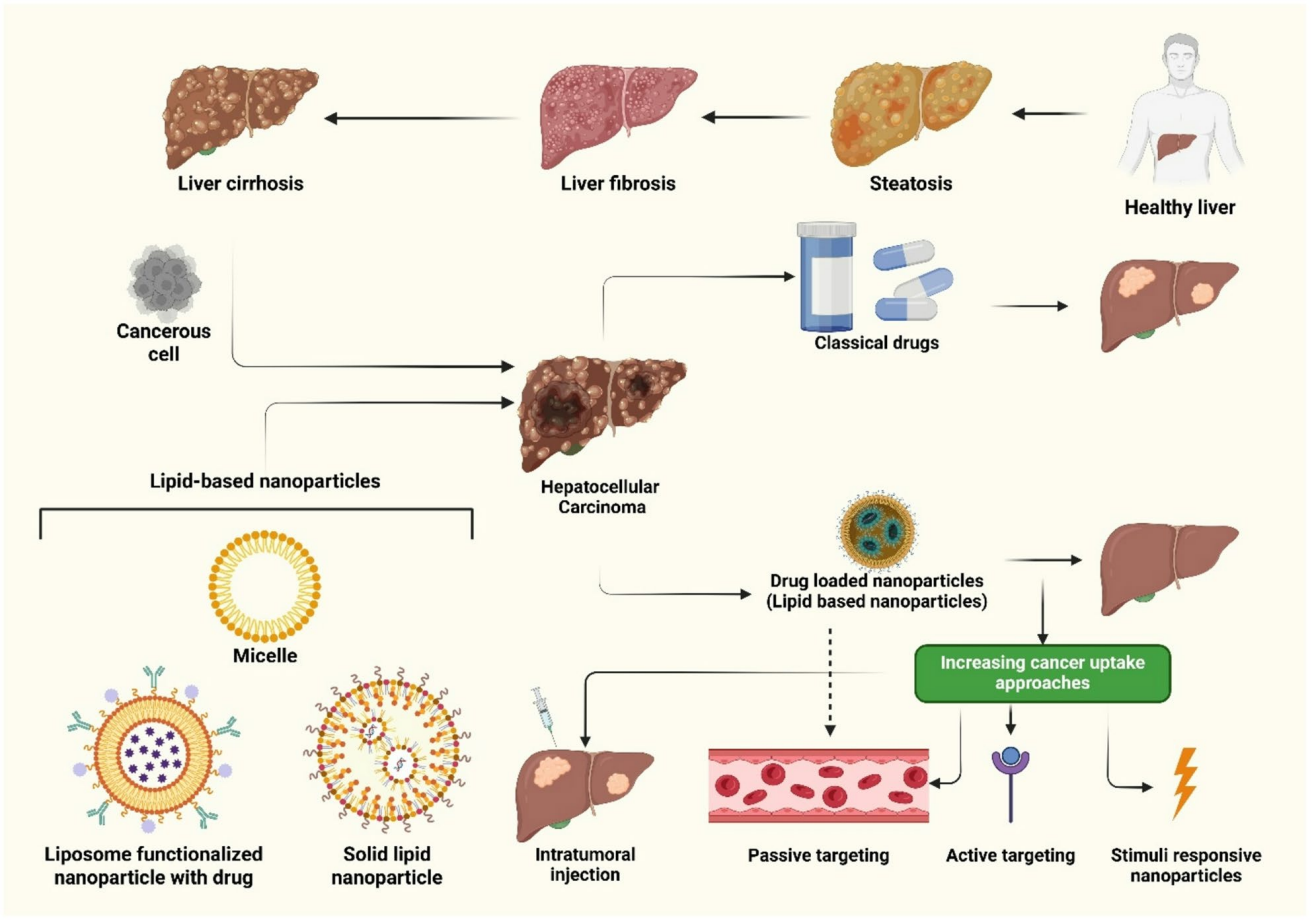
It demonstrates the progression of liver disease to HCC and highlights treatment approaches using nanotechnology. The transition from a healthy liver to HCC includes cirrhosis, fibrosis, and steatosis. Nanoparticles, including micelles and drug-loaded liposomes, enhance drug delivery and efficacy compared to conventional medicines, which often have low effectiveness. These nanocarriers facilitate targeted drug delivery, improving cancer uptake and reducing off-target toxicity through methods such as intratumoral injection and receptor-mediated targeting

HCC treatment primarily involves hepatic resection, liver transplantation, and ablative therapies. Approved first-line drugs, lenvatinib and sorafenib, only moderately improve survival. The study focused on identifying new therapeutic targets, using hydrodynamic transfection of AKT1 and β-catenin to develop HCC models. Flow cytometry revealed that 14.4% of HCC patients had co-activated Akt/mTOR and Wnt/β-catenin pathways, correlating with lower survival rates. Cells exhibiting stem-like characteristics were identified and associated with treatment resistance. Jak/Stat pathway inhibitors effectively reduced tumor growth in Akt/β-catenin-driven HCC, indicating a potential new treatment strategy to address drug resistance [39]. Both healthy and cancerous cells rely on the JAK/STAT signaling system for essential biological functions. Aberrant activation of JAK/STAT signaling in a portion of HCC leads to dysregulation of downstream target genes that lead to survival, angiogenesis, stemness, immune surveillance, invasion, and metastasis [40]. A study focuses on the anticancer properties of quercetin and the development of novel nanocarriers to enhance its effectiveness against HCC, while minimizing drug resistance. Quercetin shows potential therapeutic benefits through mechanisms such as ROS regulation, apoptosis induction, tumor cell cycle inhibition, and overcoming chemotherapy resistance. Pharmaceutical engineering efforts aim to form targeted nanocarriers that improve quercetin’s bioavailability and therapeutic impact against HCC, addressing challenges related to drug resistance [79]. Simvastatin (STAT) and pioglitazone (PIO) were evaluated in a study aimed at improving their efficacy against chemoresistant HCC using a nanotechnology-based co-delivery strategy. Lipid NPs (LNPs) were formed from chitosan (CPSLNPs) and folate-conjugated chitosan (FCPSLNPs) to encapsulate PIO and STAT. Key physicochemical properties such as size, zeta potential, and polydispersity were characterized, and safety and cytotoxicity were evaluated through hemolysis and MTT assays. Gene expression analysis indicated that PSLNP formulations significantly upregulated BAX and downregulated IL-1β, IL-6, and BCL2 in HepG2 cells. Molecular docking studies revealed complementary interactions of PIO with proteins like COX-2 and MMP-9, and STAT with SDH and JNK3. Ultimately, the functionalized PSLNPs showed potential in overcoming multidrug resistance (MDR) and enhancing the therapeutic efficacy of PIO and STAT for HCC treatment [80]. Another study highlights the role of STAT3 in HCC progression and its resistance to treatment. It developed stabilized modified antisense oligonucleotides (STAT3 ASOs) targeting STAT3 mRNA, resulting in decreased STAT3 levels in HCC cells and a significant reduction in cancer cell growth, survival, migration, and invasion. In an HCC xenograft model, STAT3 ASOs lowered tumor burden and improved the effectiveness of sorafenib, particularly against sorafenib-resistant HCC cells. STAT3 ASOs are a promising therapeutic strategy for HCC and similar malignancies exhibiting STAT3 dependence [81]. Alternative solutions to drug-related restrictions involve nanoformulations like nanoquercetin (nQ). This approach is particularly promising for HCC, where cargo-targeted delivery of nQ via extracellular vesicles (EVs) can synergistically impact several pathways, including NF-κB, p53, and JAK/STAT. The study explores signaling pathway checkpoints for potential nQ-infused modified EVs to develop a treatment for HCC. Additionally, the role of EVs in regulating miRNA expression in the HCC was investigated [82]. A study investigates the role of the IL-6-mediated JAK-STAT oncogenic signaling pathway in HCC and evaluates the effects of artesunate on this condition. In a model induced by nitrosodiethylamine (200 mg/kg), rats exhibited increased tumor markers and liver weight, indicating tumorigenesis potential. Artesunate (25 mg/kg) was effective in reducing these pathophysiological, biochemical, and immunohistochemical changes. Immunoblot analysis showed elevated levels of IL-6 and other oncogenic factors in rats treated with nitrosodiethylamine, which were reversed with artesunate supplementation, demonstrating its anti-tumor and pro-apoptotic properties through the modulation of the JAK-STAT signaling pathway [56]. Another study investigated the effects of triptolide on hepatoma cell proliferation and apoptosis, focusing on its interaction with the JAK and STAT pathway, using the HepG2 cell line divided into control and triptolide treatment groups. Triptolide reduced cell proliferation and increased apoptosis rates in a dose-dependent manner (0.02, 0.05, and 0.10 µM), with significant results. The treatment also led to decreased expression of phosphorylated JAK1 and STAT3 proteins, correlating with higher triptolide doses. The presence of transforming growth factor-beta enhanced JAK1 and STAT3 protein expression compared to the high-dose triptolide alone [83]. Lipid-based NPs **(**Fig. 4**)** provide superior safety profiles and biocompatibility compared to other nanocarriers while facilitating liver-specific delivery through mechanisms like ApoE-mediated uptake. This study focuses on siRNA therapies for HCC, stressing NP-based delivery methods, cell signaling targets, production techniques, and the role of AI in optimizing siRNA formulation and therapy customization [84]. HepG2 cell assays and in vivo studies indicate that DEHP may enhance tumor growth in HepG2 cells and HCC mice via the JAK2/STAT3 pathway [85]. Cucurbitacin E exhibits antiproliferative and apoptotic effects on HepG2 HCC cells. It has anticancer effects both alone and combined with sorafenib, a standard treatment for HCC [86]. HPssPT exhibited significant antitumor immunity in animal models for HCC, making these tumors more responsive to PD-1 inhibition and paving the way for siRNA-based immunotherapy innovations [87]. Interferon γ (IFNγ) significantly influences tumor development, with alterations in miRNA expression impacting gene regulation during carcinogenesis. Additionally, SOCS3 was confirmed as a direct target of miR-1291, facilitating the activation of the JAK/STAT pathway. These results interplay between immune responses and HCC development through miRNA regulation [88]. The compounds using nanotechnology are being designed for drug delivery systems aimed at treating HCC (Table 1).

**Table 1:** Compounds are used to prevent and treat HCC

Compounds	Loaded nanoparticles	Finding	Ref.
Cisplatin and Curcumin	Liposomes	HCC treatment with co-loaded cisplatin and curcumin nano-liposomes.	[89]
Curcumin	Curcumin-PLGA-PEG/chitosan NPs	Reduced angiogenesis, migration, and proliferation, while it stimulated the proapoptotic pathway, leading to cell death.	[90]
Curcumin and berberine	Liposomes	Treated HCC by preventing interaction between hepatic stellate cells and tumor cells.	[91]
Berberine	Janus gold mesoporous silica nanocarriers	Improved chemotherapy, radiotherapy, and photothermal treatment in liver cancer management, while also preventing radiation-induced damage.	[92]
Berberine	Mesoporous silica	CM-ss-MONs are proposed as ideal drug carriers to support the therapeutic application of berberine in liver cancer treatment.	[93]
Ganoderic acid	Nano-lipidic	Ganoderic acid nano-lipid carriers on cancer signaling pathways are investigated to improve the hepatic condition associated with diethyl-nitrosamine-induced HCC in Wistar rats.	[94]
Curcumin	Polyamidoamine dendrimer	Developed nanoscale G4 PAMAM dendrimers, involved galactosamine and loaded with the anticancer curcumin derivative, as a targeted drug delivery system for HCC.	[95]
Epigallocatechin-3-Gallate	Chitosan	The development of chemotherapeutic agents for HCC is facilitated by the finding that NPs influence the expression of genes related to apoptosis and proliferation.	[96]
Epigallocatechin-3-Gallate	Polymeric NPs	Demonstrated that the combination of AFP-siRNA and showed significant cytotoxic effects on HepG2 cells.	[97]

## Stimuli-responsive nanodrug systems for pathway-specific targeting in HCC

Stimuli-responsive biomaterials enhance HCC management by allowing precise control over drug distribution and release, improving treatment efficacy and minimizing adverse effects. Biomaterials can be developed to respond to external stimuli, such as light and magnetic fields, as well as internal cues like pH and enzymes. The integration of these materials could enhance drug transport efficiency and address biological barriers in HCC therapy, potentially revolutionizing treatment paradigms and significantly improving patient outcomes [98]. A study demonstrates a novel all-in-one therapeutic nanoplatform (FTY720@AM/T7-TL) for HCC utilizing nanomedicine. This platform combines tetraphenylethylene, fingolimod (FTY720), hybrid-liposome, gold-manganese dioxide NPs, and T7 peptides. It features a pH-responsive charge-reversal mechanism, enhancing cellular uptake and prolonging nanodrug circulation in blood. Utilizing pH and near-infrared light as stimuli facilitates controlled drug release and enhances oxygen availability in the tumor microenvironment. The platform shows significant photothermal conversion, imaging capabilities, and synergistic anti-tumor effects in tests, indicating strong potential for dual-modal guided therapy in HCC [99]. Glycolytic reprogramming in HCC plays a critical role in promoting tumor growth and developing resistance to treatment. A stimuli-responsive nanozyme incorporating glucose oxidase was developed within a pH-sensitive lipid-gelatin-protamine nanogel. This nanozyme reduces intratumoral glucose under acidic conditions, increasing the sensitivity of HCC cells to apoptosis through TRAIL and doxorubicin. Co-delivery of glucose oxidase and doxorubicin promotes immunogenic cell death and activates immune responses by enhancing dendritic cell development and CD8 + T cell activation. The nanozyme modifies the immunosuppressive environment, as shown by transcriptomic analysis, and effectively reduces tumor growth and metastasis in orthotopic HCC models, especially in combination with an anti-PD-1 checkpoint inhibitor, while not causing systemic toxicity. A multifunctional strategy demonstrates treatment resistance in HCC by integrating immunological activation with metabolic interventions [100]. HCC treatment faces challenges like side effects and anoxic medication resistance. A targeted nano drug delivery system, REG/YC-1@PTP-RGD NPs (RYP-RGD NPs), was developed to co-deliver the hypoxia inhibitor YC-1 and REG. YC-1 downregulated HIF-1α, enhanced cytotoxicity against HCC cells, and exhibited effective tumor targeting in vitro. In vivo experiments demonstrated decreased toxicity and a significant reduction in tumor growth when compared to single agents. RYP-RGD NPs provide a new strategy for HCC treatment by improving drug targeting, enhancing therapeutic efficacy, and ensuring biosafety [101]. A novel nano-platform utilizing phototherapy and small-molecule targeted therapy was developed. This platform involves loading Sorafenib and Chlorin e6 (Ce6) into hollow mesoporous manganese dioxide NPs, optimized for biocompatibility. The mesoporous manganese dioxide NPs facilitate tumor microenvironment stimuli-responsive drug release, enhancing photodynamic therapy effectiveness and reducing tumor hypoxia. In trials, mesoporous manganese dioxide NPs demonstrated significant anticancer effects on both sorafenib-sensitive and resistant HCC cells, with tumor inhibition rates of 53.4% without and up to 100% with laser irradiation. This innovative nano-platform shows promise for multimodal imaging and treatment of HCC, particularly in overcoming sorafenib resistance [102].

## Nanomaterials and advanced drug delivery platforms for the theranostic management of HCC

Emerging therapies and theranostic nanodrug delivery systems present challenges for researchers in liver cancer treatment, particularly concerning multiple drug resistance, high clearance rates, and adverse effects. Conventional chemotherapy is limited by poor drug delivery to cancer cells, need for new nanocarriers that improve targeted and sustained release of therapies. These innovative systems enhance drug concentration at tumor sites while improving cellular absorption and reducing toxicity to healthy tissues. The study demonstrates recent advancements in nanocarrier-based drug delivery systems for liver cancer, specifically excluding clinical advancements in theranostics and the limitations of single-nanocarrier systems [103]. Nanomaterial-based theranostics combines diagnosis and treatment, enhancing hepatic cancer care through targeted drug delivery, improved imaging, and real-time monitoring. Pharmaceutical sciences and nano-biomedicine focus on stimuli-responsive NPs that release therapies within the tumor microenvironment. This approach minimizes side effects and optimizes outcomes, improving patient quality of life. Innovative techniques for managing hepatic cancer are demonstrated, highlighting their potential for clinical translation [104]. In cancer theranostics, the co-administration of hydrophilic (doxorubicin) and hydrophobic (docetaxel) drugs via a novel amphiphilic PCL-AuNC/Fe(OH)3-PAA Janus NP is crucial for overcoming drug resistance and minimizing side effects. This study found sequential drug release from a single inorganic Janus NP, demonstrating a 5% improvement in therapeutic impact. Enhanced computed X-ray tomography/magnetic resonance imaging capabilities of the Janus NPs indicate their effectiveness in guiding cancer treatment. Mice treated with dual drug-loaded Janus NPs under near infrared laser irradiation showed greater tumor reduction compared to groups treated with single, cocktail, or dual drugs, underscoring the efficacy of combined cancer therapy [105]. In nanomedicine, chitosan NPs serve as effective polymeric carriers due to their mucoadhesive, biodegradable, biocompatible, and penetration-enhancing properties. Current treatments for HCC face challenges such as high recurrence rates and severe side effects, demonstrating the need for better targeting solutions. Chitosan’s surface properties facilitate the formation of NPs for targeted drug delivery and diagnostics in liver tumors [106]. The multifunctional micellar nanodrug enhances the delivery and efficacy of sorafenib for advanced HCC and aids non-invasive tumor detection through MRI. The nanodrug comprises a diblock copolymer containing sorafenib and superparamagnetic iron oxide NPs, decorated with anti-glypican-3 antibodies for targeted delivery. Its small size, weak positive charge, and active targeting improve cellular absorption and tumor accumulation. The nanodrug exhibits dual sensitivity to glutathione and lysosomal acidity, facilitating the rapid release of sorafenib into cancer cells. This approach enhances the anticancer effects of sorafenib and shows the potential of the micellar nanodrug as a theranostic device for HCC management [107].

## Toxicity and long-term safety of nanocarriers in HCC therapy

The field of medication delivery is developing with the formation of nanocarriers that enhance drug delivery systems. These nanocarriers can improve bioavailability, enable controlled and targeted release, and ultimately increase medication efficacy while reducing toxicity [108]. Doxorubicin transdrug, a NP formulation of doxorubicin, was effective in overcoming chemoresistance in HCC during preclinical studies. Its safety and efficacy were subsequently evaluated in phase I and randomized phase II trials for patients with unresectable HCC. Phase I trials established the maximal tolerated dose of doxorubicin transdrug at 30 mg/m2, with observed haematological and respiratory toxicities at elevated doses [109]. Lipid nanocapsules are demonstrated for their internalization and toxicity effects in HepG2 and HepaRG cell lines. 50 nm lipid nanocapsules had slower internalization than 100 nm lipid nanocapsules and were more toxic to cancerous HepG2 cells than to differentiated HepaRG cells. The primary internalization pathway was caveolin-mediated endocytosis. Prolonged exposure increased toxicity in HepaRG cells, causing cell death primarily through ferroptosis rather than apoptosis. 100 nm lipid nanocapsules showed a good safety profile and increased effectiveness against liver cancer cells due to quicker internalization following both acute and repeated exposures [110]. The liver can accumulate NPs via Browicz-Kupffer cells and liver sinusoidal endothelial cells, leading to the generation of harmful ROS and inflammatory cytokines. Strategies to reduce hepatotoxicity include PEGylation of liposomes and graphene NPs, negatively charged lipids, charge manipulation, protein-based NPs for cancer targeting, and size adjustments. It is demonstrated that pH-responsive drug release modifications in liposomes and graphene NPs to tackle hepatotoxicity in approved therapies [111]. The dose-limiting toxicities of siRNA nanocarriers are influenced by Kupffer cells’ release of PAF, which promotes inflammation and affects vascular signaling. High doses of siRNA-polymer nano-polyplexes (si-NPs) in mice led to symptoms akin to shock, correlated with increased plasma PAF and decreased PAF acetylhydrolase (PAF-AH) activity. Preventive measures such as PAF receptor inhibition or depletion of Kupffer cells eliminated these effects. The toxicity of various si-NP chemistries is linked to the absorption by liver Kupffer cells, which was notably higher in tumor-bearing mice. Kupffer cell PAF release is a key factor in siRNA nanocarrier toxicity and suggests that PAFR inhibition could enhance tolerance to higher siRNA nanocarrier doses [112].

## Clinical trials and patents

Clinical trials for HCC are challenging due to the prevalence of liver disease among patients, necessitating high-quality trials. The American Association for the Study of Liver Diseases has set guidelines for HCC trial design, focusing on the necessity of randomized phase 2 trials that utilize time-to-event endpoints, such as time to progression. While survival is a key measure in phase 3 trials, endpoints like progression-free and disease-free survival may inadequately reflect clinical benefits. Standard treatments like sorafenib and chemoembolization are recommended for control groups. Integration of biomarkers and molecular imaging is essential for improving trial efficacy, with a recommendation for specialized centers to spearhead these initiatives [113]. Mitoxantrone-loaded polybutylcyanacrylate NPs (DHAD-PBCA-NPs) demonstrate enhanced cytotoxicity in hepatic tumors. In a study of unresected HCC patients, the DHAD-PBCA-NPs showed an objective response rate of 10.5%, with 28.1% exhibiting progression and 61.4% maintaining stable disease. In the DHAD injection group, there was no objective response; 54.9% experienced disease progression, while 45.1% had stable disease. Significant differences in disease progression and stability were observed. Median survival was 5.46 months for DHAD-PBCA-NPs versus 3.23 months for DHAD injection. Leukopenia affected 47.4% of the DHAD-PBCA-NPs arm and 74.5% of the DHAD injection arm, while anemia occurred in 37.3% of the former and 65% of the latter [114]. A wound-targeted nanodrug, referred to as a-PM-S-MSNP, combines an immune checkpoint inhibitor (anti-PD-L1) and the angiogenesis inhibitor sorafenib using biocompatible mesoporous silica NPs coated with platelet membrane. In a murine orthotopic HCC model, a-PM-S-MSNP showed a significant anti-HCC effect and enhanced longevity in immune-competent mice. Immunophenotyping demonstrated positive anti-HCC immunity and anti-angiogenesis in tumor sites, with two of six treated mice showing no tumors histologically, in contrast to the control group. The study indicates the potential of a-PM-S-MSNP for clinical application to prevent post-surgical HCC relapse without adverse side effects [101]. A pragmatic randomized clinical trial involving 1800 cirrhosis patients evaluated the effectiveness of mailed outreach and patient navigation on HCC surveillance. Participants were assigned to three groups: mailed outreach for screening ultrasound, mailed outreach with patient navigation, and usual care. During 18 months, HCC surveillance rates were 23.3% for the outreach/navigation group, 17.8% for the outreach-alone group, and 7.3% for the routine care group. The combination of outreach and navigation improved the surveillance rate by 5.5% over outreach alone and by up to 16% compared to usual care (*P* < 0.001). Early detection rates were similar among groups, but a greater percentage of patients with screen-detected HCC presented with early-stage tumors (83.3% compared to 30.8%, *P* = 0.003). Mail outreach and navigation improve HCC surveillance in cirrhosis patients more effectively than standard care [115]. Between 2017 and 2020, the FDA approved several new therapies, including immune checkpoint inhibitors (nivolumab and pembrolizumab) and multikinase inhibitors (regorafenib, lenvatinib, cabozantinib, and ramucirumab), along with bevacizumab combined with atezolizumab. Over 1000 active clinical trials are investigating therapies for HCC, indicating a vibrant research environment. FDA-approved agents for HCC are demonstrated, with a focus on promising compounds from clinical phase I/II/III trials and emerging treatment targets for HCC [116]. Clinical development of systemic therapies for HCC has increased in recent years, especially after disappointing phase 3 trial results for PD1-inhibitors like nivolumab and pembrolizumab. Initial positive outcomes from atezolizumab and bevacizumab as first-line treatments are anticipated to significantly alter HCC management and improve the potential for additional combination therapies. Recent developments in clinical trials for systemic treatments of HCC include ongoing studies on adjuvant and neoadjuvant therapies, as well as first and second-line treatments for advanced and intermediate HCC cases [117]. HCC is challenging to treat due to its diverse origins. This study analyzes patents related to HCC treatments to identify advancements that may guide research, R&D prioritization, and policymaking. Utilizing the Derwent Innovation platform, a systematic review of 528 patent families and 2543 documents identified eleven technological classifications, emphasizing molecular target therapy, chemotherapy, locoregional therapy, combination therapies, and immunotherapy. Advancements in targeted therapies include antibody-drug conjugate technology, drug-eluting beads for trans-artery chemoembolization, and progress in the Notch pathway. Emerging trends in molecular therapy emphasize novel targets, enhanced locoregional therapies, and the integration of combined immunotherapy with targeted therapies [118]. HCC contributes to cancer-related deaths, with limited late-stage treatments and inadequate early detection methods. Innovative nanotechnology is being explored to tackle these challenges in diagnosis and treatment. Recent patents indicate progress in the field, particularly with new HCC biomarkers and enhanced imaging techniques like Ga3 + and nanogold CT contrast agents. Nanodrugs, utilizing polymer-based NPs and liposomal formulations, enhance drug efficacy while minimizing required dosages [119].

## Conclusion and future perspectives

HCC presents a significant therapeutic challenge due to its aggressive growth, molecular heterogeneity, and limited response to traditional therapy. Aberrant NF-κB and JAK/STAT signaling significantly contribute to angiogenesis, immune evasion, tumor-associated inflammation, hepatocarcinogenesis, and resistance to systemic therapies. Nanotechnology-based drug delivery methods are revolutionizing treatment approaches for HCC by addressing existing limitations. This review discusses significant advancements in nanocarriers designed to modulate JAK/STAT and NF-κB signaling. It highlights various nanoplatforms, including polymeric systems, inorganic nanomaterials, lipid-based NPs, and biomimetic carriers, which enhance drug solubility, prolong systemic circulation, increase tumor-specific accumulation, and facilitate controlled or stimulus-responsive drug release. Furthermore, to maximize treatment design and patient classification, a systems-level understanding of the intricate interactions between the JAK/STAT and NF-κB pathways and other oncogenic and immune-regulatory networks is required. Validating the efficacy and safety of nanotherapeutics in HCC patients necessitates precisely planned clinical trials and specific regulatory structures. Targeting the NF-κB and JAK/STAT pathways with nanotechnology may significantly improve the treatment and outcomes of HCC. HCC treatments may be transformed by nanomedicine through the application of artificial intelligence (AI) and machine learning (ML). These technologies can enhance NP design by predicting physicochemical properties, drug loading efficiency, and targeting accuracy. ML enables personalized nanotherapeutics by identifying patient-specific biomarkers, while AI predicts pharmacokinetics, biodistribution, and toxicity, reducing the duration of in vivo studies. The combination of AI with high-throughput screening and imaging methods enhances drug delivery precision, driving precision oncology in HCC treatment and improving clinical translation and therapeutic outcomes.

## Data Availability

No datasets were generated or analysed during the current study.

## Abbreviations

HCC: Hepatocellular carcinoma
NF-κB: Nuclear factor kappa-light-chain-enhancer of activated B cells
JAK: Janus kinase
STAT: Signal transducer and activator of transcription
IL: Interleukin
IL-6: Interleukin-6
HBV: Hepatitis B virus
HCV: Hepatitis C virus
MAFLD: Metabolic-associated fatty liver disease
VEGF: Vascular endothelial growth factor
MRI: Magnetic resonance imaging
EMT: Epithelial–mesenchymal transition
TME: Tumor microenvironment
OS: Oxidative stress
ROS: Reactive oxygen species
MAPK: Mitogen-activated protein kinase
PI3K: Phosphoinositide 3-kinase
Akt: Protein kinase B
IFN: Interferon
IFN-α: Interferon alpha
IFN-γ: Interferon gamma
SOCS: Suppressor of cytokine signaling
miRNA / miR: MicroRNA
circRNA: Circular RNA
siRNA: Small interfering RNA
PDX: Patient-derived xenograft
NPs: Nanoparticles
PLGA: Poly(lactic-co-glycolic acid)
PEG: Polyethylene glycol
LNPs: Lipid nanoparticles
ASO: Antisense oligonucleotide
TLR: Toll-like receptor
EGFR: Epidermal growth factor receptor
RFA: Radiofrequency ablation
RCC: Renal cell carcinoma
HFSR: Hand-foot skin reaction
TKIs: Tyrosine kinase inhibitors
ICIs: Immune checkpoint inhibitors
MDR: Multidrug resistance
EASL: European Association for the Study of the Liver
RECIST: Response Evaluation Criteria in Solid Tumors
GNPs: Potential of gold nanoparticles
ESRD: End-stage renal disease
CKD: Chronic kidney disease
PCA: Portulacerebroside A
TMC-Cys: Trimethyl chitosan–cysteine
HepG2: Human hepatocellular cancer cells
